# Cell membrane capacitance relationship to reference-measured body composition parameters in young adult athletes

**DOI:** 10.2478/joeb-2025-0015

**Published:** 2025-10-14

**Authors:** Dale R. Wagner

**Affiliations:** Kinesiology & Health Science, Utah State University, Logan, UT, USA

**Keywords:** Bioimpedance, fat-free mass index, phase angle, reactance, resistance

## Abstract

Cell membrane capacitance (Cm) is considered a measure of cellular health. This study evaluated the relationship between bioimpedance spectroscopy-measured Cm and multicomponent model reference-measured body composition variables from air displacement plethysmography and dual-energy x-ray absorptiometry in a sample of 226 young adult athletes. Men (3.00 ± 0.62 nF) had greater (*p* < 0.001) Cm than women (1.90 ± 0.36 nF). Variables indicative of lean mass, such as fat-free mass index, had a strong (*r* > .70) direct relationship with Cm. The Cm relationship was moderate for measures related to body mass and bone health (*r* = .30 to .60) and weak (*r* < .20) for fat mass. The relationship between Cm and body composition variables is strongest for the fat-free components.

## Introduction

Interest in the biological impedance of an electrical current dates back over 100 years [1], but contemporary use of bioelectrical impedance analysis (BIA) to estimate fat-free mass (FFM) [2,3] and total body water (TBW) [4] began in earnest in the mid-1980s. Evidence of the continued use of this technology for estimating body composition is a recent 2024 review [5] summarizing 106 BIA prediction equations. Beyond body composition assessment, impedance technology is used for the clinical assessment of hydration status, fluid accumulation, wound healing, and disease prognosis [6].

Impedance (Z) to electrical current is a function of resistance (R) and reactance (Xc), such that Z^2^ = R^2^ + Xc^2^. Both R and Xc are measured with BIA. Resistance is the opposition to current flow; Xc is the opposition to current flow due to cell membrane capacitance (Cm), or the ability of the lipid bilayer cell membrane to store the electrical charge [7]. Over the past two decades, there has been a growing interest in the related variable phase angle (PhA), which represents the delay between the current and voltage drop at the cell membrane; PhA = arctangent of (Xc/R) × 180°/π [8]. A higher PhA is thought to reflect greater cellularity and cell membrane integrity, whereas a lower PhA suggests a more permeable cell membrane and is associated with aging and various disease states [9].

Single-frequency BIA operates at 50 kHz; consequently, PhA is measured at 50 kHz. In contrast, bioimpedance spectroscopy (BIS) operates at hundreds of frequencies. Using BIS, a Cole plot of Xc (y-axis) and R (x-axis) results in a semi-circular curve with the high point of the curve called the characteristic frequency. This frequency is used to calculate Cm (measured in nanofarads; nF). In healthy adults, the characteristic frequency is typically close to 50 kHz; thus, PhA and Cm, although different values with different units of measurement, are both surrogate measures of “cellular health”. See the review by Ward and Brantlov [10] for a more detailed explanation of the relationship between PhA and Cm.

The published literature on PhA is extensive, and age-, sex-, and BMI-specific PhA reference values exist [11]. In addition, the relationship of PhA to other body composition parameters has been reviewed [12]. In contrast, Cm is rarely discussed in the literature. The aim of this brief report is to examine the relationship between Cm and body composition variables collected from a multicomponent analysis. A sample of young adult athletes provides a healthy baseline of Cm values that can serve as a preliminary reference for future Cm studies. Given the similarity between PhA and Cm, the hypothesis is that Cm will be directly related to measures of lean mass and TBW, but less related to other body composition variables.

## Materials and methods

### Participants

This Cm dataset came from a multicomponent model study of university club sport athletes [13] in which TBW was estimated from BIS. Consequently, all of the participants were university club sport athletes. Exclusion criteria included current pregnancy, a pacemaker or other implanted cardiac device, surgical metal implant, and loss of limb.

### Procedures

Height was measured to the nearest 0.1 cm with a wall-mounted stadiometer (Seca 222, Seca Corp., Ontario, CA). Body mass was measured to the nearest 0.01 kg with a Bod Pod scale (Cosmed USA, Inc., Concord, CA). Body volume and bone mineral content were measured with air displacement plethysmography (ADP) (Bod Pod, Cosmed USA, Inc., Concord, CA) and dual-energy x-ray absorptiometry (DXA) (Horizon-W, Hologic, Inc., Marlborough, MA), respectively. Details of these procedures are provided elsewhere [13].

An SFB7 BIS machine (ImpediMed, Inc., Carlsbad, CA) was used to obtain the Cm values and an estimate of TBW. Prior to testing, the machine was calibrated with the manufacturer-provided test cell. Participants were supine for 5 min before measurement. During this time, hair was shaved in the areas of electrode placement if necessary, and the area was cleaned with an alcohol wipe. Electrodes were placed in a tetrapolar configuration on the right side of the body with proximal electrodes bisecting the styloid processes at the wrist and malleoli at the ankle. The manufacturer’s dual-tab electrodes were used to ensure that the distal electrodes were 5 cm from the proximal electrodes. Two readings were taken. Post-collection, the manufacturer’s Bio-imp software (version 5.5.0.1) was used to collect the Cm values; the average of the two readings was used for subsequent analysis.

### Statistical analyses

Fat mass was calculated from the Wang et al. [14] four-component formula, and other body composition parameters such as FFM and body fat percentage were derived from this value. Descriptive statistics were calculated as mean ± SD. Sex difference for Cm was evaluated with an independent *t*-test. The relationships between Cm and body composition parameters of interest were quantified with Pearson correlation coefficients. Qualitative judgment of the strength of correlation was made using the values suggested by Hopkins et al. [15]: .1 (small or weak), .3 (moderate), .5 (large or strong), .7 (very large/strong), and .9 (extremely large/strong). Statistical analyses were done using SPSS version 27 (IBM, Inc., Armonk, NY).

### Informed consent

Informed consent was obtained from all individuals included in this study.

### Ethical approval

The research related to human use complied with all relevant national regulations, institutional policies and the tenets of the Helsinki Declaration, and was approved by the authors’ institutional review board.

## Results

The descriptive characteristics of the 226 athletes (38.9% women) who completed the data collection are in Table 1. A more detailed analysis of this sample, such as body composition by sport, is available elsewhere [13]. The Cm of men (3.00 ± 0.62 nF) was significantly greater (*p* < 0.001) than that of women (1.90 ± 0.36 nF). A sex difference (*p* < 0.001) remained even when corrected for body mass (Cm/mass). Pearson correlation coefficients between Cm and body composition variables collected from a multicomponent model analysis are in Table 2.

**Table 1.** Descriptive characteristics of the study sample. Data are means ± SD.

**Variable**	**Men** (*n* = 138)	**Women** (*n* = 88)	**Total** (*N* = 226)
Age (y)	21.3 ± 1.8	20.2 ± 1.7	20.9 ± 1.9
Height (cm)	179.4 ± 6.8	166.6 ± 7.1	174.4 ± 9.3
Mass (kg)	79.8 ± 12.5	66.6 ± 11.7	74.7 ± 13.8
BMI (kg/m^2^)	24.8 ± 3.6	23.9 ± 3.5	24.4 ± 3.5
Body volume (L)	74.9 ± 12.5	64.0 ± 12.1	70.7 ± 13.4
Body density (g/cc)	1.0663 ± 0.0162	1.0429 ± 0.0150	1.0572 ± 0.0194
Fat percent (%)	14.3 ± 5.8	25.2 ± 6.0	18.6 ± 7.9
Fat mass (kg)	11.8 ± 6.5	17.3 ± 7.1	14.0 ± 7.2
TBW (L)	48.5 ± 6.7	35.4 ± 4.6	43.4 ± 8.8
TBW/mass (%)	61.1 ± 4.1	53.6 ± 4.2	58.2 ± 5.5
BMC (kg)	3.034 ± 0.403	2.328 ± 0.308	2.759 ± 0.504
BMD (g/cm^2^)	1.294 ± 0.101	1.174 ± 0.072	1.247 ± 0.108
Fat-free mass (kg)	68.0 ± 8.7	49.4 ± 6.1	60.7 ± 11.9
FFMI (kg/m^2^)	21.1 ± 2.3	17.8 ± 1.7	19.8 ± 2.7
Cm (nF)	3.00 ± 0.62	1.90 ± 0.36	2.58 ± 0.76

BMI = body mass index, TBW = total body water, BMC = bone mineral content, BMD = bone mineral density, FFMI = fat-free mass index, Cm = membrane capacitance

**Table 2.** Correlation coefficients for membrane capacitance (Cm) with measured body composition parameters. The 95% confidence interval is in parentheses.

Variable	Men (*n* = 138)	Women (*n* = 88)	Total (*N* = 226)
Height	.001	.046	.485
	(−.166 – .168)	(−.166 – .255)	(.378 – .579)
Mass	.556	.423	.651
	(.429 – .662)	(.233 – .582)	(.568 – .720)
BMI	.609	.490	.478
	(.492 – .705)	(.311 – .635)	(.370 – .573)
Body volume	.540	.409	.601
	(.410 – .649)	(.217 – .570)	(.511 – .679)
Body density	−.227	−.206	.294
	(−.379 – −.062)	(−.399 – .005)	(.169 – .409)
Fat percentage	.025	.044	−.459
	(−.143 – .191)	(−.168 – .252)	(−.556 – −.349)
Fat mass	.179	.201	−.143
	(.012 – .336)	(−.010 – .395)	(−.268 – −.012)
TBW	.726	.645	.860
	(.637 −.797)	(.502 – .753)	(.822 – .891)
TBW/mass	.197	.119	.561
	(.031 – .352)	(−.094 – .321)	(.464 – .645)
BMC	.410	.265	.679
	(.261 – .540)	(.058 – .451)	(.601 – .744)
BMD	.304	.266	.557
	(.145 – .449)	(.058 – .451)	(.460 – .641)
Fat-free mass	.667	.585	.837
	(.563 – .751)	(.427 – .709)	(.793 – .872)
FFMI	.776	.719	.859
	(.700 – .835)	(.599 – .807)	(.821 – .890)

BMI = body mass index, TBW = total body water, BMC = bone mineral content, BMD = bone mineral density, FFMI = fat-free mass index

## Discussion

With the exception of height, variables of body size (mass, BMI, body volume) had a moderate to strong positive relationship with Cm when grouped by sex. The relationship between fat (fat mass, body fat percentage, body density) and Cm was trivial to weak, whereas Cm was moderately positive with bone (bone mineral content, bone mineral density) and strongly to very strongly related to measures of lean mass (TBW, FFM, fat-free mass index [FFMI]). The highest correlation with Cm was with FFMI, and FFMI is indicative of how much muscle mass an athlete is carrying on his/her skeletal frame [16]. Collectively, the correlations suggest the larger the individual and the more muscle mass that person is carrying, the higher the Cm, but fat mass is less influential to Cm.

The results found in the present investigation are consistent with other researchers. In a sample of 60 premenopausal women, Barry et al. [17] reported a stronger correlation with Cm for lean mass measured by DXA (.65) than fat mass (.45) or body fat percentage (.32). The mean Cm for women in the present study was higher than the 1.58 ± 0.47 nF reported by Barry et al. [17], and this corresponds to the present study sample being younger and more athletic with more muscle mass.

Given the similarity between Cm and PhA, it is not surprising that the pattern of relationship between Cm and body composition variables parallels that of PhA and body composition parameters. A review of PhA concluded that there was a direct relationship between PhA and lean mass or FFM in 86% of the studies reviewed; however, the relationship between fat mass and PhA was equivocal with a nearly equal number of studies reporting a direct relationship, inverse relationship, and no relationship [12]. In a systematic review of the PhA of athletes [18] there was a positive correlation between PhA and BMI similar in strength to the relationship between Cm and BMI in the present study.

The utility of knowing one’s Cm value is still unclear to researchers. Cm is thought to be a measure of cell integrity [10]; consequently, Cm should be related to health and performance. A suggested mechanism for this relationship is that Cm is highly indicative of transverse tubule development (i.e., cell membrane surface area) in skeletal muscle, and this corresponds with greater maximum voluntary contraction [19]. Variables such as muscle quality and cell integrity are not measurable with other body composition methods such as DXA and ADP, highlighting the potential practical application of measuring Cm with BIS.

Results from the present study are limited to young adult athletes. One should expect lower Cm values for children and older adults. A strength of this investigation was the use of reference methods to measure the body composition variables. Martins et al. [12] noted in their scoping review of the relationship between PhA and body composition that few studies used reference methods to measure the body composition components. In conclusion, men have a greater Cm than women. Further, there is a very strong direct relationship between Cm and measures of lean mass, particularly FFMI, but the correlation between Cm and fat mass is weak.

## References

[1] Ward LC (2021). Electrical bioimpedance: from the past to the future. J Electr Bioimp.

[2] Lukaski HC, Johnson PE, Bolonchuk WW, Lykken GI (1985). Assessment of fat-free mass using bioelectrical impedance measurements of the human body. Am J Clin Nutr.

[3] Segal KR, Gutin B, Presta E, Wang J, Van Itallie TB (1985). Estimation of human body composition by electrical impedance methods: a comparative study. J Appl Physiol.

[4] Kushner RF, Schoeller DA (1986). Estimation of total body water by bioelectrical impedance analysis. Am J Clin Nutr.

[5] Campa F, Coratella G, Cerullo G, Noriega Z, Francisco R, Charrier D (2024). High-standard predictive equations for estimating body composition using bioelectrical impedance analysis: a systematic review. J Transl Med.

[6] Lukaski HC (2013). Evolution of bioimpedance: a circuitous journey from estimation of physiological function to assessment of body composition and a return to clinical research. Eur J Clin Nutr.

[7] Kushner RF (1992). Bioelectrical impedance analysis: a review of principles and applications. J Am Coll Nutr.

[8] Foster KR, Lukaski HC (1996). Whole-body impedance – What does it measure?. Am J Clin Nutr.

[9] Norman K, Stobaus N, Pirlich M, Bosy-Westphal A (2012). Bioelectrical phase angle and impedance vector analysis – Clinical relevance and applicability of impedance parameters. Clin Nutr.

[10] Ward LC, Brantlov S (2023). Bioimpedance basics and phase angle fundamentals. Rev Endocr Metab Disord.

[11] Bosy-Westphal A, Danielzik S, Dorhofer R-P, Later W, Wiese S, Muller MJ (2006). Phase angle from bioelectrical impedance analysis: population reference values by age, sex, and body mass index. J Parenter Enteral Nutr.

[12] Martins PC, Alves Junior CAS, Silva AM, Silva DAS (2023). Phase angle and body composition: a scoping review. Clin Nutr ESPEN.

[13] Wagner DR, Heath EM, Harper SA, Cafferty EA, Teramoto M, Evans A (2025). Multicomponent body composition of university club sport athletes. J Int Soc Sport Nutr.

[14] Wang ZM, Deurenberg P, Guo SS, Pietrobelli A, Wang J, Pierson RN (1998). Six-compartment body composition model: inter-method comparisons of total body fat measurement. Int J Obes.

[15] Hopkins WG, Marshall SW, Batterham AM, Hanin J (2009). Progressive statistics for studies in sports medicine and exercise science. Med Sci Sports Exerc.

[16] Jagim AR, Harty PS, Jones MT, Fields JB, Magee M, Smith-Ryan AE (2024). Fat-free mass index in sport: normative profiles and applications for collegiate athletes. J Strength Cond Res.

[17] Barry VG, Chiang JL, Bowman KG, Johnson KD, Gower BA (2023). Bioimpedance-derived membrane capacitance: clinically relevant sources of variability, precision, and reliability. Int J Environ Res Public Health.

[18] Di Vincenzo O, Marra M, Scalfi L (2019). Bioelectrical impedance phase angle in sport: a systematic review. J Int Soc Sports Nutr.

[19] Yamada Y, Hirata K, Iida N, Kanda A, Shoji M, Yoshida T (2022). Membrane capacitance and characteristic frequency are associated with contractile properties of skeletal muscle. Med Eng Phys.

